# Correcting for non-participation bias in health surveys using record-linkage, synthetic observations and pattern mixture modelling

**DOI:** 10.1177/0962280219854482

**Published:** 2019-06-11

**Authors:** Linsay Gray, Emma Gorman, Ian R White, S Vittal Katikireddi, Gerry McCartney, Lisa Rutherford, Alastair H Leyland

**Affiliations:** 1 MRC/CSO Social and Public Health Sciences Unit, University of Glasgow, Glasgow, UK; 2 Department of Economics, Lancaster University, Lancaster, UK; 3 MRC Clinical Trials Unit at UCL, London, UK; 4 Directorate of Public Health and Health Policy, NHS Lothian, Edinburgh, UK; 5 NHS Health Scotland, Glasgow, UK; 6 ScotCen Social Research, Edinburgh, UK

**Keywords:** Missing not at random, multiple imputation, non-participation, pattern-mixture modelling, record-linkage, survey data

## Abstract

Surveys are key means of obtaining policy-relevant information not available from routine sources. Bias arising from non-participation is typically handled by applying weights derived from limited socio-demographic characteristics. This approach neither captures nor adjusts for differences in health and related behaviours between participants and non-participants within categories. We addressed non-participation bias in alcohol consumption estimates using novel methodology applied to 2003 Scottish Health Survey responses record-linked to prospective administrative data. Differences were identified in socio-demographic characteristics, alcohol-related harm (hospitalisation or mortality) and all-cause mortality between survey participants and, from unlinked administrative sources, the contemporaneous general population of Scotland. These were used to infer the number of non-participants within each subgroup defined by socio-demographics and health outcomes. Synthetic observations for non-participants were then generated, missing only alcohol consumption. Weekly alcohol consumption values among synthetic non-participants were multiply imputed under missing at random and missing not at random assumptions. Relative to estimates adjusted using previously derived weights, the obtained mean weekly alcohol intake estimates were up to 59% higher among men and 16% higher among women, depending on the assumptions imposed. This work demonstrates the universal value of multiple imputation-based methodological advancement incorporating administrative health data over routine weighting procedures.

## 1 Introduction

Population health and health behaviour estimates are commonly derived from survey data to monitor trends and formulate and evaluate policies. However, bias may arise if the survey samples are not representative of the target population. Non-representativeness is of some concern when measures of association such as relative risk are being estimated^1^ but of greater concern for population prevalence and quantity estimates,^2–4^ such as for alcohol consumption.^5^ A key aspect influencing the extent to which surveys are representative is the level of non-participation (unit non-response) among individuals included in the sampling frame. For instance, there is likely to be a group of harmful and dependent drinkers who may be disinclined to participate.

Survey weights derived from inverse probability weighting^6^ are usually applied in an attempt to correct for such unit non-response (as well as accounting for aspects of sampling design such as the oversampling of certain household types or geographical areas). However, these weights typically rely on a limited range of socio-demographic variables^7^ and are based on the assumption that non-participants have equivalent behaviours to participants in the same socio-demographic category which is unlikely to be the case.

An alternative to the application of survey weights is multiple imputation (MI),^8^ which is viable if the assumption that data are missing at random (MAR: the probability of missingness is unrelated to the unobserved data conditional on the observed data) holds. Alanya et al. applied MI to make unit non-response adjustments and compared it to weighting.^9^ They found MI to compare favourably, though not consistently so. In another comparison with weighting, MI showed comparable performance in terms of bias but also yielded substantially lower variance estimates.^10^ However, these papers made no allowance for the data being missing not at random (MNAR: the probability of missingness is related to the unobserved data). If the data are thought to be MNAR then an alternative approach is required, typically involving sensitivity analyses, and using methodology such as pattern mixture modelling^11^ among others.^12,13^

Application of MI is strengthened if we can infer information on the absent non-participants. In the absence of whole population registers, as existing in Nordic countries,^14^ nations typically lack individual-level data amenable to forming the bases of sampling frames. Thus, in countries such as the UK, individual non-participants cannot readily be identified and their routine health data extracted.

We propose a novel methodology that aims to improve addressing non-participation bias in national health survey data in order to obtain less biased estimates of alcohol consumption.^15,16^ We consider both MAR and MNAR within a missing data framework, motivated by the possibility of non-participants differing in their alcohol consumption^17^ from survey participants with the same socio-demographic variables and health outcome statuses. Our approach involves: (1) exploitation of record-linkage to hospital discharges and mortality; (2) survey–population comparisons which inform the creation of synthetic partial observations for non-participants; and (3) MI to generate refined estimates of weekly consumption of alcohol under assumptions of MAR (weaker than when based on survey data alone) and explorations of MNAR.^18^ We illustrate the application using data from the 2003 Scottish Health Survey (SHeS) individually record-linked to administrative health information from the Scottish Morbidity Records (SMR), mortality data from the National Records of Scotland (NRS) and unlinked contemporaneous data for the entire population.

In the next section, we provide the context and motivation for the methodological approach described in section 3. In section 4, we report on the application before discussing the implications in section 5 and concluding in section 6.

## 2 Motivating example and data

### 2.1 Aim

We aim to devise and apply methodology to estimate sex-specific adult population mean alcohol consumption from national health survey data accounting for bias induced by non-participation.

### 2.2 SHeS

SHeS are a series of cross-sectional surveys designed to represent the Scottish population living in private households.^19^ Socio-demographic data available in the surveys include sex, age group and Scottish Index of Multiple Deprivation (an area-based measure of deprivation collapsed into five equal population-weighted groups), collectively referred to here as ‘socio-demographic characteristics’. Alcohol consumption is calculated in units (equivalent to 10 ml or 8 g of pure ethanol) per week. Pre-derived survey sampling weights which sum to the achieved sample total have been created to account for the stratified, multi-stage random sample survey design and departures from population estimates by sex and age.^19^ We use the 2003 survey which had an adult response level of 60%.

### 2.3 Linked health outcomes

**Figure 1.**
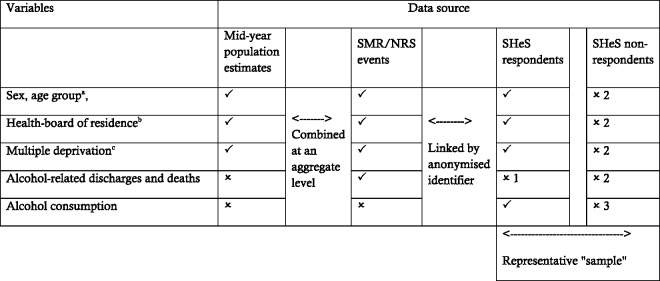
Available data from mid-year population estimates, Scottish Morbidity Records/National Records of Scotland, Scottish Health Survey data sources and desired data on SHeS non-respondents.

Baseline data on consenting SHeS participants (91%) have been confidentially linked to routinely-collected nationwide administrative health records available until the end of 2011 providing prospective follow up of around eight years. These include prospective SMR which record hospital discharges (∼90% accurate diagnosis, 99% complete^20^) and mortality data using a probabilistic matching algorithm^21–24^ (Figure 1).

### 2.4 Population data

For the general population, mid-year population estimates – available by sex, age group and area deprivation – for 2003 were used as denominators.^25^ Numerator counts of morbidity and mortality events in the population during the eight years of follow-up were combined with mid-year population estimates – also by socio-demographic characteristics – to create an unlinked aggregate-level data set for the population for comparison with the record-linked survey data.

Two pertinent binary ‘health outcome’ variables were created from the morbidity and mortality data, the first indicating hospitalisation or death from an alcohol-related cause during the follow-up period (taken together as comprising alcohol-related harm, Supplemental Table 1) and the second indicating all-cause mortality during follow-up. The analyses were restricted to individuals aged 20 to 64 years in the survey year in an attempt to reduce the distortion of institution-dwelling communities (e.g. older people living in care homes) – which are not in the sampling frame – on the survey-population comparisons.

**Table 1.** Sex- and area deprivation group-specific breakdowns (%) for the general population of Scotland and participants^a^ in the Scottish Health Survey 2003 aged 20 to 64 years consenting to linkage with inferred estimates for non-participants.

Area deprivation group	Population		Scottish Health Survey					
			Respondents		Synthetic non-respondents		Inferred total	
	Men (%)	Women (%)	Men (%)	Women (%)	Men (%)	Women (%)	Men (%)	Women (%)
Least deprived	10.4	10.5	10.6	11.4	10.2	9.5	10.4	10.5
2	10.0	10.2	10.2	10.9	9.8	9.6	10.0	10.3
3	9.7	10.0	9.1	9.7	10.6	10.4	9.8	10.0
4	9.6	10.2	9.7	10.3	9.5	10.1	9.6	10.2
Most deprived	9.1	10.1	8.4	9.9	10.0	10.4	9.1	10.1
*All groups*	48.9	51.1	47.9	52.1	50.1	49.9	48.9	51.1

a Those participants consenting to record-linkage of their data.

## 3 Methodology

**Figure 2.**
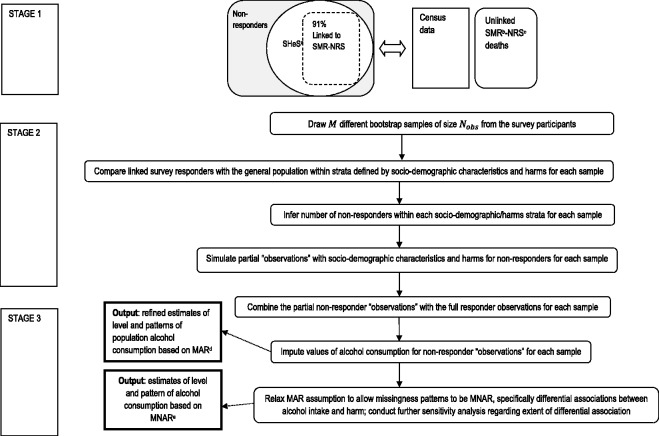
Summary of methodological strategy for addressing survey non-representativeness and refining alcohol consumption estimates. ^a^SHeS: Scottish Health Survey; ^b^SMR: Scottish Morbidity Record; ^c^NRS: National Records of Scotland; ^d^MAR: missing at random; ^e^MNAR: missing not at random.

Our approach to addressing non-participation bias in alcohol consumption estimates involved filling in the missing data in the survey in three stages marked as 1, 2 and 3 in Figure 1. The three stages are depicted in Figure 2 and described in detail in sections 3.2 to 3.4, with a worked example given in section 4.1. We compare the results of our approach with those obtained from the traditional survey-weighted results.

### 3.1 Notation

We use the following notation. Let $N_{\mathrm{obs}}$ be the number of linkage-consenting participants (henceforth referred to as ‘participants’ for simplicity of terminology) in the survey and let $N_{\mathrm{mis}}$ be the converse ‘non-participants’ (comprising those who did not participate and those who did participate but did not consent to record linkage). Let *L* denote the effective response level – the percentage of the sample who both responded to the survey and consented to linkage (determined by the product of the survey response level and the consent proportion and henceforth referred to as ‘response level’). We infer the total survey size to be $N=N_{\mathrm{obs}}/L$. Let X be the set of categorical random variables for the socio-demographic covariates; here *X*_1_ is sex (1 for men and 2 for women), *X*_2_ is age group and *X*_3_ is area deprivation quintile. Let H be the set of random variables for the health outcomes; here *H*_1_ corresponds to alcohol-related harm, and *H*_2_ corresponds to all-cause mortality. Let *Y* denote the random variable for usual weekly alcohol consumption as a semi-continuous measure (detailed in section 3.4.1).

Let *S* denote the binary random variable for the source of the data, with S=SHeS for the target survey sample (i.e. participants and non-participants combined) and S=Pop for the administrative data. For the survey data, let *R* be the binary random variable for response, such that R=1 for survey participants and R=0 for survey non-participants. Pre-derived sampling weights for the survey participants are denoted *w* and have a mean of one. The observed linked survey data are therefore (RX,RY,RH) while the unobserved data are ((1-R)X,(1-R)Y,(1-R)H). Let [.] denote a distribution. Finally, let *M* be the total number of repeated independent data sets arising from MI.

### 3.2 Stage 1: Using record linked data

In stage 1, record linkage of survey data to SMR and NRS data was used to determine the values of H for consenting survey participants. Surveyed individuals who did not give consent to record-linkage were treated as non-participants i.e. their survey data observations were excluded, as this was deemed a pragmatic approach.

### 3.3 Stage 2: Creation of synthetic observations for non-participants

In stage 2, we made inference on the non-participants by comparing the national health survey data with corresponding population data to identify deviations from representativeness in terms of H in addition to X. We generated $N_{\mathrm{mis}}=N-N_{\mathrm{obs}}$ synthetic observations for the non-participants to the SHeS and filled in their values of X and H, as follows.

We first assumed that [X,H] – which we know from the population data – is the same in the target survey sample (irrespective of *R*) as in the population

A1⋮[X,H|S=SHeS]=[X,H|S=Pop]

This assumption is valid if the sampling frame for the survey is representative of the general population.

It follows that the X and H characteristics of non-participants can be inferred by comparison of survey and general population data, exploiting the categorical nature of X and H. Using the equality

$$\begin{array}{c} P(X,H|S=\mathrm{SHeS})=P(X,H|S=\mathrm{SHeS},R=1)P(R=1|S=\mathrm{SHeS})\\ +P(X,H|S=\mathrm{SHeS},R=0)P(R=0|S=\mathrm{SHeS}) \end{array}$$

we can write, using A1

(1) $$P(X,H|S=\mathrm{SHeS},R=0)=\frac{P(X,H|S=\mathrm{Pop})-P(X,H|S=\mathrm{SHeS},R=1)P(R=1|S=\mathrm{SHeS})}{P(R=0|S=\mathrm{SHeS})}$$

where each term on the right hand side can be estimated from the data: for estimation of P(X,H|S=SHeS,R=1), simple weighted prevalences for each combination of X and H were used; P(R=1|S=SHeS) is *L* (for which weighting may be ignored). We hence identified the number of missing participants within each socio-demographic group in the survey as $N_{\mathrm{mis}}\times P(X,H|S=\mathrm{SHeS},R=0)$. The corresponding number of non-participant synthetic observations with assigned characteristics were generated for each (X,H) category, with usual weekly alcohol consumption, *Y*, set to missing, and *w* set to 1. Combining the participants with the synthetic observations for the non-participants provides a data set for imputation.

Three modifications were needed to this general method. First, the method as proposed does not allow for uncertainty in the survey-based estimates of P(X,H|S=SHeS,R=1) arising from sampling variation. To accommodate such uncertainty, we drew *M* different bootstrap samples of size $N_{\mathrm{obs}}$ from the survey participants, yielding *M* different imputed datasets. Theoretically, the bootstrap method could have been extended to allow for uncertainty in P(X,H|S=Pop) and P(R=1|S=SHeS), but we did not do this since these quantities were estimated with little imprecision.

Second, the calculated numbers of missing participants in each category were generally not integers. To avoid possible bias due to rounding, we applied random rounding which preserves the mean count. For example, if 2.6 missing participants were required in a particular category, then we took 3 missing participants with probability 0.6, and 2 missing participants with probability 0.4. This was performed separately in each imputed data set.

Third, estimates of P(X,H|S=SHeS,R=0) are in some instances negative due to sampling variation in cells with small numbers. This was handled by removing synthetic observations in the nearest neighbouring category or categories: for example, if a particular category $(X,H)=(x_{1},x_{2},x_{3},h_{1},h_{2})$ required -3 synthetic observations, we identified the nearest category of synthetic individuals and randomly deleted 3 of them. The metric defining distance was the sum of the squared differences between the values of categories $(X,H)=(x_{1},x_{2},x_{3},h_{1},h_{2})$ scaled by the squared standard deviation.

### 3.4 Stage 3: Imputing alcohol consumption for non-participants

Once the synthetic observations for the non-participants were created at Stage 2, the unit (person) non-response problem had been converted into an item (variable) non-response problem with the synthetic non-participant observations having data on socio-demographic characteristics and health outcomes but missing data on alcohol consumption. Imputation models for alcohol consumption could then be specified conditional on socio-demographic characteristics and health outcomes. Missing alcohol consumption observations among a small minority of participants (*n*_ _= 16) were imputed in the same way as for non-participants.

The imputation approach we used begins by assuming that, given the fully observed data on health outcomes as well as socio-demographic characteristics, non-participation in the SHeS-SMR data set is MAR (note that this is already an improvement on standard methods based on unlinked data, for which MAR would not condition on health outcomes). We then accommodated the possibility of the data being MNAR by allowing the distribution of alcohol consumption to differ in a pre-specified manner between the non-participants and participants (given the fully observed characteristics including health outcomes). Sections 3.4.1 and 3.4.2 outline in turn the MI procedures based on MAR and MNAR.

For both MAR- and MNAR-based approaches, one stochastic imputation was performed for each of the *M* data sets of synthetic non-participant observations produced in stage 2, ultimately yielding *M* multiply imputed data sets. The imputed data sets were appended and weighted substantive analyses were performed using -mim-^26^ in Stata 13.1 (StataCorp, Texas).

#### 3.4.1 MI assuming MAR

Under MAR, conditional on socio-demographic characteristics and health outcomes, the distribution of alcohol consumption is independent of participation status. This is assumption **A2**:

A2⋮[Y|X,H,S=SHeS,R=0]=[Y|X,H,S=SHeS,R=1]

*Y* is a semi-continuous variable characterised by a combination of zeros representing those who do not report drinking and a right skewed continuous distribution of positive consumption. We followed a previously adopted approach^27^ to handle the nontrivial proportion of zero values of alcohol consumption by using a two-part model for *Y*, splitting it into the dichotomous drinking status variable *D* and continuous consumption variable $Y_{i}^{*}$, where

(2) $$\begin{array}{c} D=\{\begin{array}{c} 1\text{ if }\;Y>0\\ 0\text{ if }\;Y=0 \end{array} \end{array}$$

(3) $$\begin{array}{c} Y^{*}=\{\begin{array}{c} g(Y)\text{ if }\;D=1\\ \mathrm{undefined}\text{ if }\;D=0 \end{array} \end{array}$$

Testing of the log transformation g(y)=log(y) led to a left-skewed distribution of $Y^{*}$ (a nontrivial proportion of values for alcohol consumption between 0 and 1 unit per week) and hence some extreme imputed values. Instead we used g(y)=log(y-k), the shifted log transformation^28^ with a shift parameter, *k*, selected to eliminate the skew of $Y^{*}$ ($\overset{\wedge }{k}$_ _= −1.9, 95% CI: −2.3 to −1.6). Predictive mean matching with a potential match pool of size 10 was used to further improve the imputations.

Our model for [Y|X,H,S=SHeS,R=1] is therefore

(4) $$\begin{array}{c} logitp(D=1)=\alpha_{D}+\beta_{D}X+\gamma_{D}H+\zeta_{D}w\;\\ Y^{*}|D=1,X,H\;∼\;N(\alpha_{Y}+\beta_{Y}X+\gamma_{Y}H+\zeta_{Y}w,\sigma^{2}) \end{array}\}$$

where $\alpha_{D},\beta_{D},\gamma_{D},\zeta_{D},\alpha_{Y},\beta_{Y},\gamma_{Y},\zeta_{Y},\sigma$ are regression parameters estimated from the complete data. These models were specified jointly in Stata using the conditional imputation option of -ice-,^29^ within strata defined by sex and deprivation group to allow the association between harms and alcohol consumption to vary flexibly by sex and deprivation level. The *w* were entered as a continuous predictor in both equations in equation (4) as well as being included as sampling weights to account for survey design.^30^ This procedure produced imputed data sets which allowed correctly for uncertainty in the parameters $\left(\alpha_{D},\beta_{D},\gamma_{D},\zeta_{D},\alpha_{Y},\beta_{Y},\gamma_{Y},\zeta_{Y},\sigma \right)$. Imputed values were back-transformed for use in the substantive model for *Y*.

#### 3.4.2 MI assuming MNAR

We sought to change imputations of *Y* to reflect plausible differences between participants and non-participants in *Y* given a range of theoretically informed plausible departures from the MAR assumption. We did so by specifying how the conditional distribution of *Y* differs between participants and non-participants and hence altering the imputation model^31^ in sensitivity analyses using a pattern mixture approach.^11^

We embed the MAR model in a wider class of models containing sensitivity parameters.^32,33^ The sensitivity parameters describe the difference in the joint distribution of fully observed data on participants and partially observed data on non-participants. Under MAR, the joint distribution [Y|X,H] is assumed equivalent for participants and non-participants alike according to assumption **A2**, whereas under MNAR, **A2** is relaxed.

Pattern mixture modelling offers a means to model the joint probability distribution of *Y* and *R*, allowing the distribution of *Y* to differ depending on whether or not *Y* is observed (equation (5)). Differences between the conditional distributions [Y|X,H,S=SHeS,R=0] and [Y|X,H,S=SHeS,R=1] are specified by a set of sensitivity parameters δ. These differences cannot be identified from our data themselves without making untestable distributional assumptions or parameter restrictions.^34^ One way of tackling this is to specify the values based on plausible hypotheses about differences between participants and non-participants,^31^ with reference to external data or expert opinion.^35^ Examples of reference sources are given in section 3.4.3.

Our principal rationale for exploring MNAR concerns differential overall drinking levels, but the possibility remains that *D* may also deviate from MAR (for instance, for reasons such as lack of social cohesion, non-participants may have a different chance of drinking given their socio-demographics and health outcomes statuses). To assess the sensitivity of results to deviations from the MAR assumption for *D*, two categories of scenarios were considered for the imputation of *D*: first, imputing under a MAR assumption, and second, under an ‘upper bound’ scenario in which all non-participants are set as drinkers for comparison. The rationale for this being of more substantive interest is that those who do not respond in any given sociodemographic and harm group are more rather than less likely to drink, and that it gives an interval which we can be certain about even if we lack a specific plausible deviation from MAR. We then modified the imputation procedure for $Y^{*}$ as detailed in section 3.4.3.

We consider the general specification which accommodates differential modification of the imputation model by *H*_1_ and by a modifying variable, in our case $X_{1}.$ Our choice of model is governed by the trade-off between increasing model accuracy against increased difficulty in eliciting plausible δ values

(5) $$Y^{*}|D=1,X,H,R∼N(\alpha_{Y}+\beta_{Y}X+\gamma_{Y}H+\zeta_{Y}w+(1-R)(\delta_{0}^{X_{1}}(1-H_{1})+\delta_{1}^{X_{1}}H_{1}),\sigma^{2})$$

Relative to participants with fully observed alcohol consumption, mean alcohol consumption is modified by $\delta_{0}^{X_{1}}$ among non-participants who do not experience alcohol-related harms, depending on *X*_1_; and similarly by $\delta_{1}^{X_{1}}$ among the non-participants who experienced alcohol-related harms. Clearly, MAR is the case for which $\delta_{0}^{X_{1}}=\delta_{1}^{X_{1}}=0$ for all *X*_1_.Various scenarios for the magnitudes and signs of the δ parameters are considered in section 3.4.3.

#### 3.4.3 Specifying the parameters governing deviations from MAR

We considered two general approaches to specifying possible values for parameters $\delta_{0}^{X_{1}}$ and $\delta_{1}^{X_{1}}$. The first uses specific SHeS ‘paradata’ on fieldwork effort to secure participation (MNAR1 and MNAR2) and the second draws on existing literature-based subject-matter knowledge (MNAR3, MNAR4 and MNAR5). Within each of these, we explored both the MAR (e.g. MNAR1M) and ‘upper bound’ (e.g. MNAR1UB) approaches for imputing the drinking status (see footnote to Table 4 for full notation).

- ***Survey paradata-based approach***

We drew on continuum-of-resistance theory which is predicated upon the idea of a latent propensity to not participate.^2,3,36,37^ Here, invited households who do not initially respond are re-approached one or more times, and the number of interviewer calls is recorded. Later responding participants can be theorised to be increasingly more like non-participants with the greater effort required to recruit them into the survey. We used the number of interviewer calls to a household as our proxy for non-participation propensity, where an individual who responded in three (the median number) or fewer attempts is considered an early-participant, and those that took four or more attempts are considered late-participants. Estimates of $\delta_{0}^{X_{1}}$ and $\delta_{1}^{X_{1}}$ are derived by estimating the mean difference in consumption between early- and late-participants among those who experience alcohol-related harms and those who do not, separately by sex, adjusting for age group and deprivation group. Taking the differences in consumption between early- and late-participants to inform us on the differences between participants and non-participants in this way is speculative in the absence of a more direct proxy but can be thought to represent a conservative MNAR estimate (MNAR1M). Setting the deviation from MAR to be equal to the adjusted difference between early- and late-participants resulted in values for $\delta_{0}^{X_{1}=1}$_ _= 1.0 and $\delta_{1}^{X_{1}=1}$_ _= 23.1 among men, and $\delta_{0}^{X_{1}=2}$_ _= 0.75 and $\delta_{1}^{X_{1}=2}$_ _= 1.17 among women, respectively.

We also considered the scenario in which the consumption deviation from MAR is twice the adjusted difference between early- and late-participants (MNAR2M and MNAR2UB).

- ***Literature-based approach***

A second form of sensitivity analysis considered a range of deviations from the MAR specification based on subject-matter knowledge. A survey in Scotland specifically sampled harmful and dependent drinking in-patients and out-patients attending alcohol addiction services in two Edinburgh hospitals, finding an estimated mean weekly consumption of 198 (95% CI: 185–211) units.^38^ For our purposes we posit this to be a generalisable estimate of consumption among drinkers who have been hospitalised. We therefore considered the MNAR-based sensitivity analysis where the imputation model involves specifying $\delta_{1}^{X_{1}}$ such that that the resulting overall mean weekly consumption, among those experiencing alcohol-related harm, would equal approximately 198 units. This corresponds to a scenario where the deviation from MAR is five-times the observed sex-specific mean among those whose experienced harm ($\delta_{1}^{X_{1}=1}$_ _= 309.7, $\delta_{1}^{X_{1}=2}$_ _= 91.8; denoted MNAR5M and MNAR5UB). We also considered more moderate scenarios, where the deviation from MAR consumption was three-times the observed sex-specific mean among those whose experienced harm ($\delta_{1}^{X_{1}=1}185.8,\delta_{1}^{X_{1}=2}$_ _= 55.1; MNAR4M and MNAR4UB;) and finally where the deviation from MAR was equal to the observed sex-specific mean among those whose experience harm ($\delta_{1}^{X_{1}=1}$_ _= 62.0, $\delta_{1}^{X_{1}=2}$_ _= 18.3; MNAR3M and MNAR3UB). $\delta_{0}^{X_{1}=1}$_ _= $\delta_{0}^{X_{1}=2}$_ _= 0 in all these scenarios.

## 4 Application

### 4.1 Non-participant synthetic observations (Stage 2)

The SHeS had an overall survey response level of 60% and a proportion of consent to record linkage in Stage 1 of 0.91 with $N_{\mathrm{obs}}=$5381 participants aged 20 to 64 years consenting to linkage. This yielded an effective response level, L=54.6%. We therefore estimated the total number of participants which would have been observed under full response as N= 9855 and the number of non-participant synthetic observations to be generated in Stage 2 as $N_{\mathrm{mis}}=$4474. We chose to draw *M*_ _= 70 bootstrap samples to be imputed, based on the fraction of missing information of 70%.^39^

As a numerical example, consider the category of (X,H) defined by men, aged between 40 and 44, residing in the most deprived area quintile, who in 2003–2011 were admitted to hospital with an alcohol-related diagnosis but did not die (i.e., $H_{1}=1$, and $H_{2}=0$). In this category, P(X,H|S=Pop)_ _= 0.001135, and using the first bootstrap sample, P(X,H|S=SHeS,R=1)_ _= 0.001021. Since also P(R=1|S=SHeS)_ _= 0.546, P(R=0|S=SHeS)_ _= 0.454, equation (1) gives P(X,H|S=SHeS,R=0)_ _= 0.001272, and $N_{\mathrm{mis}}\times P(X,H|S=\mathrm{SHeS},R=0)$_ _= 5.691381. This figure was randomly rounded up to 6.

After the creation of the synthetic observations, the combined samples were largely successful in reflecting the desired (population representative) socio-demographic composition and health outcome probabilities (Tables 1, 2 and 3).

**Table 2.** Eight-year probabilities of alcohol-related harm in the population, in the Scottish Health Survey participants^a^ and the synthetic non-participants in 2003 by sex and area deprivation group.

	Population		Scottish Health Survey					
			Respondents		Synthetic non-respondents		Inferred total	
Area deprivation group	Men (%)	Women (%)	Men (%)	Women (%)	Men (%)	Women (%)	Men (%)	Women (%)
Least deprived	1.9	0.9	1.2	0.7	2.7	1.3	1.9	0.9
2	3.0	1.4	1.3	1.6	4.9	1.2	2.9	1.5
3	4.3	2.0	3.6	2.1	5.0	1.8	4.2	1.9
4	6.6	2.8	4.2	2.2	9.3	3.4	6.5	2.8
Most deprived	11.4	4.2	5.8	2.6	16.4	5.9	11.1	4.2
*All groups*	5.4	2.3	3.1	1.8	7.6	2.8	5.2	2.2

a Those participants consenting to record-linkage of their data.

**Table 3.** Eight-year probabilities of all-cause mortality in the general population, in the Scottish Health Survey participants^a^ and the synthetic non-participants in 2003 by sex and area deprivation group.

	Population		Scottish Health Survey					
			Respondents		Synthetic non-respondents		Inferred total	
Area deprivation group	Men (%)	Women (%)	Men (%)	Women (%)	Men (%)	Women (%)	Men (%)	Women (%)
Least deprived	2.5	1.7	1.1	1.4	3.7	2.1	2.2	1.7
2	3.3	2.2	2.4	1.8	4.2	2.5	3.2	2.1
3	4.4	2.8	2.6	1.7	5.7	3.6	4.1	2.6
4	5.7	3.5	3.8	2.3	6.8	4.2	5.1	3.1
Most deprived	8.3	4.5	7.6	4.6	7.4	3.4	7.5	4.1
*All groups*	4.8	2.9	3.3	2.3	5.6	3.2	4.4	2.7

a Those participants consenting to record-linkage of their data.

**Table 4.** Weekly alcohol consumption estimates in the Scottish Health Survey 2003 participants^a^ and the ‘full sample’ by sex under various assumptions about the missing data.

	Men					Women				
	$\delta_{0}$	$\delta_{1}$	Mean	(95% CI)	% change	$\delta_{0}$	$\delta_{1}$	Mean	(95% CI)	% change
Survey-weighted	–	–	21.8	(20.5–23.1)	–	–	–	10.8	(10.1 –11.6)	–
MAR	–	–	22.4	(20.3–24.4)	+3	–	–	10.8	(9.8–11.7)	0
*Survey paradata-based approach*										
MNAR1M	1.0	23.1	23.7	(21.8–25.6)	+9	0.75	1.17	11.1	(10.1–12.0)	+3
MNAR2M	2.0	26.2	24.9	(22.8–27.0)	+15	1.50	2.34	11.5	(10.5–12.4)	+7
MNAR1UB	1.0	23.1	25.0	(22.4–27.5)	+15	0.75	1.17	11.7	(10.6–12.7)	+9
MNAR2UB	2.0	26.2	26.2	(23.6–28.8)	+20	1.50	2.34	12.0	(11.0–13.1)	+11
*Literature-based approach*										
MNAR3M	0.0	62.0	24.6	(22.4–26.7)	+13	0.0	18.3	11.0	(10.0–12.0)	+2
MNAR4M	0.0	186	28.9	(26.4–31.5)	+33	0.0	55.1	11.5	(10.5–12.5)	+7
MNAR5M	0.0	310	33.3	(30.1–36.5)	+53	0.0	91.8	11.9	(10.8–13.0)	+10
MNAR3UB	0.0	62.0	25.9	(23.2–28.6)	+20	0.0	18.3	11.6	(23.2–28.6)	+7
MNAR4UB	0.0	186	30.3	(27.2–33.3)	+40	0.0	55.1	12.0	(10.9–13.1)	+11
MNAR5UB	0.0	310	34.7	(31.1–38.3)	+60	0.0	91.8	12.5	(11.4–13.6)	+16

a Those participants consenting to record-linkage of their data. $\delta_{0}$: sex-specific missing not at random based addition to mean alcohol consumption among non-participants who do not experience alcohol-related harms; $\delta_{1}$: sex-specific missing not at random based addition to mean alcohol consumption among non-participants who experience alcohol-related harms (δ multiples appear non-exact due to rounding); CI: confidence interval; MAR: missing at random; MNAR1M: missing not at random based on survey paradata approach using the adjusted difference between early- and late-participants assuming MAR drinking status; MNAR2M: missing not at random based on survey paradata using twice the adjusted difference between early- and late-participants assuming MAR drinking status; MNAR3M: conservative literature-guided missing not at random approach to deriving delta in which deviation from MAR is equal to the observed sex-specific mean among those whose experienced harm; MNAR4M: intermediate literature-based missing not at random approach to deriving delta in which the deviation from MAR is three-times the observed sex-specific mean among those whose experienced harm; MNAR5M: literature-based missing not at random approach to deriving delta in which the deviation from MAR is five-times the observed sex-specific mean among those whose experienced harm; M: assuming MAR drinking status; UB: the upper bound in which all non-participants are classed as drinkers; %-change: percentage change from survey-weighted estimate.

### 4.2 MAR-based MI results (Stage 3)

A total of 4903 participants (91.1%) were classed as current drinkers with the remaining 478 participants (8.9%) considered non-drinkers (ex-drinkers or lifetime abstainers). Mean weekly consumption from the survey-weighted estimates was 21.8 units for men and 10.8 units for women. Imputing usual weekly alcohol consumption in Stage 3 using each of the created bootstrap sample data sets under a MAR assumption, resulted in an estimate of 22.4 units (3% increase) among men and 10.8 units (0% change) for women (MAR results in Table 4).

### 4.3 MNAR-based MI results

- ***Survey paradata-based approach***

The first scenario, in which the deviation from MAR is equal to this adjusted difference between early- and late-participants, yielded mean weekly consumption of 23.7 units among men and 11.1 units among women (Table 4, MNAR1M). For the second scenario, in which the deviation from MAR is twice the adjusted difference between early- and late-participants, the figures were 24.9 units (14% increase) and 11.5 units (6% increase), respectively. Table 4, MNAR2M). The corresponding results under the assumption that all non-participants were drinkers gives figures of 25.0 units (15% increase) for men and 11.7 units (8% increase) for women in the first scenario (Table 4, MNAR1UB) and 26.2 units (20% increase) for men and 12.0 units (11% increase) for women in the second scenario (Table 4, MNAR2UB).

- ***Literature-based approach***

Among men, adjusted mean consumption under the literature-based scenarios ranged from 24.6 units (13% increase) in the most conservative sensitivity analyses (MNAR3M) to 33.3 units (53% increase) in the most extreme (MNAR5M). Among women, this range was smaller with corresponding figures of between 11.0 units (2% increase) and 11.9 units (10% increase), respectively (Table 3, MNAR3M and MNAR5M). The corresponding results under the assumption that all non-participants were drinkers gave figures ranging from 25.9 units for men and 11.6 units for women (Table 4, MNAR3UB) to 34.7 units for men and 12.5 units for women in the second scenario (Table 4, MNAR5UB).

## 5 Discussion

Our approach forms an important additional analytic strategy for addressing non-participation in population-sampled studies. The key innovations of our approach are the incorporation of auxiliary topic-relevant data into unit non-response correction in addition to the conventional socio-demographic data, combined with the creation of synthetic observations for non-participants and the application of pattern-mixture modelling to explore sensitivity to plausible departures from the MAR assumption. Resultant alcohol consumption estimates were sensitive to assumptions regarding both drinking status of non-participants and consumption level differences between participants and non-participants. The refined estimates were between 3% and 59% higher among men and up to 16% higher among women relative to the regular survey-weighted estimates. Given that survey-based alcohol consumption estimates scale up to approximately half those indicated by sales data,^40^ our higher estimates appear to be most appropriate.

### 5.1 Strengths and limitations of this study

The first strength of this work is the utilisation of linked survey records enabling the extension of comparisons of participants and the general population from basic socio-demographic variables to health outcomes.^41^ We circumvented the challenges associated with gaining rich data characterising the population, and non-participants in particular, by generating synthetic observations for non-participants. The second strength is the application of the much discussed but little implemented ‘principled sensitivity analysis’^33^ pattern mixture modelling to optimally^42^ and transparently specify MNAR models.^43^ In cases where no delta values are obviously more realistic than others, Rubin has emphasized the need for easily communicated models^18,43^ which are particularly valued by policymakers;^44,45^ we found it useful to impose assumptions in order to fix upon a plausible mechanism, considering specific conceivable scenarios in the context of the *a priori* information available.

Limitations include the possibility of distortion arising from survey participants not consenting to record linkage which could explain some of the disparities between health outcomes in the survey samples relative to the general population; however, this only affects 9% of participants and preliminary analyses suggest minimal differences between these groups (data available on request) indicating that this is unlikely to greatly distort findings. The available alcohol-related harm outcome measures were restricted to the relatively extreme occurrences, hospitalisation and death, with no data on the more frequent occurrences of commonplace harms related to alcohol abuse such as nausea, cognitive impairment and missed working days. This may explain the relatively small changes seen in the MAR estimates despite large survey-population differences in alcohol-related harm.^46^ Previous work on refinement of alcohol consumption data in the presence of non-participation has been based on Swedish data^41^ which has also considered the implications for impact of the use of augmented data on estimates of consumption prevalence but was based on retrospective alcohol-related hospitalisation data. This offers an alternative approach which does not rely on the attendant passage of time required for follow-up data. This methodology alone is unable to address bias arising from participants mis-reporting their alcohol consumption. It is possible to account for such self-reporting bias by way of incorporation of sales data, for instance.^16^

### 5.2 Methodological strategy considerations

The following considers possible alternatives approaches in specific steps of the analyses:

- As an alternative to our procedure of generating synthetic observations and implementing MI, we could have applied weights or taken a Bayesian-based approach^47^ based on health outcome statuses as well as socio-demographic characteristics. It is not clear how to implement MNAR methods with easily communicated models in these approaches.
- A possible alternative to creating multiple data sets of synthetic non-participants followed by single stochastic imputation on each is a nested MI procedure where more than one final imputed data set is generated for each first-stage imputed data set. This could be computationally efficient if stage 2 was very slow and stage 3 was relatively fast, which was not the case here, and could help to partition the fraction of missing information between stages 2 and 3, but would require alternative combining rules to Rubin’s.^48^
- The assumptions and relative merits of our approaches to determining delta values for the pattern-mixture approach are inherently untestable, and there is an array of alternatives to the propensity- and literature-based scenarios, including: *(1) Other within-survey proxy non-participants:* e.g. those with other risky health behaviours such as heavy smoking: this may not form a useful reference point when considering plausibility of delta values; *(2) Expert opinion:*^35,49^ we canvassed the broader international alcohol research community through a mailing list (administered by the Kettil Bruun Society) to informally elicit expert opinion; no useable information was gained from this channel; *(3) Record-linked cohort data*: the use of baseline alcohol data on cohort study subjects including those who drop out during follow-up (taken as proxies for non-participants on the basis that they may be somehow similarly ‘disengaged’) and those remaining in the study follow-up (acting as the corresponding counterparts for survey participants);^50^ no such suitable data could be identified; *(4) Retail data*: such external sources of information could be used as the basis a plausible upper bound for population mean consumption; *(5) Worst-case bounds making no assumptions about the missing data*: completely assumption-free approaches, which consider all feasible values of the missing data, generate exceedingly wide bounds for continuous measurements like alcohol and are thus often not directly useful for policy purposes; approaches to narrow such bounds often rely on instrumental variables or longitudinal survey data which were not available in this case.^51^

### 5.3 Implications

National survey data are crucial resources for quantifying and monitoring trends in health related behaviours with information used for the development, implementation and evaluation of social and public health policy. As such, methodological improvements are of interest to a wide international audience of policy makers and researchers. The development of an effective post-hoc correction procedure for ever-worsening non-response in resource-intensive population-sampled studies offers an enhancement at no additional cost to data collection. This advanced methodology will potentially be applicable to existing and future surveys wherever there is the capacity to record-link surveys with administrative data. Presently, linkage of survey data to routine health records represents a cost-effective means of generating valuable longitudinal data but is performed in very few countries. In exploiting such linkage, our work demonstrates the extended utility of record linkage, providing further impetus for its wider uptake internationally.

Synthetic generation of survey non-participants is not necessary in countries with unique population identifiers and comprehensive linkage (such as the Nordic countries) with the ability to follow-up all individuals regardless of participation status.^14,52^ However, possible ethical issues related to accessing outcome data of individuals who have chosen not to participate in a survey may mean that even in such countries stage 1 of our approach might be applicable. Regardless, stages 2 and 3 of our proposed methodology would be applicable in these settings. Our approach to the sensitivity analyses was specific to the context in terms of the estimate of interest and level of participation. Different applications will require distinct approaches to be formulated. In considering the most suitable derived estimates (the higher ones, in our case), we were guided by overall estimates obtainable from external alcohol retail data; dependent on the specific context of the wider applications, reference should be made, where possible, to such relevant sources.

The presented application suggests that non-response may contribute to the general under-estimation of alcohol consumption in survey estimates. There is scope for application to other survey-derived information, which can be discrete – cigarette smoking and obesity, for instance – for which only stage 3 of our procedure would need amending. The outcomes of choice in our application were alcohol-related harms and all-cause mortality on the basis of their strong association with alcohol consumption; single or multiple outcomes can be selected and good candidate outcomes for specific applications are those which have the strongest associations with survey items of interest. Further, non-health external data sources such a taxation or education records could be used to provide auxiliary information to correct for non-participation bias in other research areas. Moreover, this paper describes tackling a single incomplete variable; however, the method can be extended to multiple incomplete variables.

### 5.4 Further work

The current method requires that the informative data for creating the synthetic non-participants are categorical, since we are determining the missing numbers within discrete cells. It may be possible to incorporate continuous data – such as the number of health outcomes experienced – in a further stage by inferring the distribution among the non-participants such that a value could be assigned to each synthetic non-participant as a draw from that distribution and repeated across multiple replications to allow properly for uncertainty. This would be most appropriately performed by way of MNAR imputation to incorporate information about number of alcohol-related harms from the population comparison data.

Sensitivity analyses could potentially be used to address any differential consumption-outcome associations among area deprivation categories, i.e. allowing for the possibility of interaction effects suggested by the greater levels of alcohol-related harm among the more deprived for equivalent levels of consumption^53^ or differential consumption-harm relationships by alcohol product type.^54^ The application we describe focussed on a quantity estimate but there is growing recognition that non-representativeness can also lead to bias in estimates of associations.^55^ We plan to develop, apply and test our methodology for association estimates.

A major alternative to the pattern-mixture approach to MNAR sensitivity analysis is the selection model approach.^56,57^ Selection modelling expresses departures from MAR as coefficients in a logistic regression model for non-participation on alcohol consumption and other covariates: the sensitivity parameter may therefore be less intuitive than in the pattern-mixture framework,^32,35^ and hence less easy to relate to subject-matter knowledge.^49^ Shared parameter models,^58^ in which the measurement of interest and missingness processes are joint modelled, offer yet another option which can be explored.

## 6 Conclusions

We offer a means to extend the addressing of non-representativeness in survey data beyond the use of conventional inverse probability weights by developing a methodology which harnesses administrative and record-linked data. The key advantage of our approach is the relaxing of the assumption that socio-demographically equivalent participants and non-participants are alike in other ways: the application of the MAR method to administrative health record-linked data is an improvement on the conventional application of survey weights, and the MNAR methods utilise the best available data to make plausible assumptions about how they might differ.

## Supplementary Material

Supplementary material

Supplementary material

Supplementary material
