# Supplementary material for: Correcting for non-participation bias in health surveys using record-linkage, synthetic observations and pattern mixture modelling
Source: Stat Methods Med Res. 2019 Jun 11;29(4):1212–26. doi: 10.1177/0962280219854482 (PMC7188518; doi:10.1177/0962280219854482)
Supplement: Supplementary material [file Supplemental_Material.pdf]

Supplemental Table 1. International Classification of Disease Classification Codes

**Alcohol-related hospital episodes**	
Prior to 01/04/1996	
ICD-9 codes (hospital episodes)	2651 (other and unspecified manifestations of thiamine deficiency);
	3039 (alcohol dependence syndrome);
	3050 (nondependent abuse of alcohol);
	2918 (other specified alcoholic psychoses);
	2910 (delirium tremens);
	2913 (other alcoholic hallucinosis);
	2915 (alcoholic jealousy);
	2919 (alcoholic psychoses, unspecified);
	2911 (korsakov's psychosis, alcoholic);
	2912 (other alcoholic dementia);
	3575 (alcoholic polyneuropathy);
	4255 (alcoholic cardiomyopathy);
	5353 (alcoholic gastritis);
	5710 (alcoholic fatty liver);
	5711 (acute alcoholic hepatitis);
	5712 (alcoholic cirrhosis of liver);
	5713 (alcoholic liver damage, unspecified);
	7607 (fetus/newborn aff. by noxious influences trans. by placenta/breast milk);
	7598 (other specified anomalies);
	7903 (excessive blood level of alcohol);
	9800 (toxic effect of ethyl alcohol);
	9801 (toxic effect of isopropyl alcohol);
	9809 (toxic effect of unspecified alcohol);
	E860 (accidental poisoning by alcohol, not elsewhere classified);
	V57 (care involving use of rehabilitation procedures);
	E9473 (adverse effects in therapeutic use of alcohol deterrents);
	E9509 (suicide and s.i. poisoning by other/unspec. solid and liquid substances);
	E9809 (poisoning undet. acc./purp. by other/unspec. solid and liquid substances);
	E8600[*] (accidental poisoning by alcoholic beverages);
	E8601[*](acc. pois. other/unspec. ethyl alcohol);
	E8609[*] (accidental poisoning by unspecified alcohol);
	E9509[*](suicide and s.i. poisoning by other/unspec. solid and liquid substances);
	5711[§] (acute alcoholic hepatitis);
	6554[§] (suspected damage to fetus from other disease in the mother);
	2550[§] (cushing's syndrome);
	3594[§] (toxic myopathy);
	5709[§] (acute and subacute necrosis of liver);
	3483[§] (encephalopathy, unspecified);
	D3039+A3317[a] (alcohol dependence syndrome+cerebral degeneration in other diseases classified elsewhere); D3039+A3344[a] (alcohol dependence syndrome+cerebellar ataxia in disease classified elsewhere).
01/04/1996 onward	

ICD-10 codes (hospital episodes)	F10 (Mental & behavioural disorders due to use of alcohol); K70 (Alcoholic liver disease);<br>X45 (Accidental poisoning by and exposure to alcohol);<br>X65 (Intentional self-poisoning by and exposure to alcohol);<br>Y15 (Poisoning by and exposure to alcohol undetermined intent);<br>Y90 (Evidence of alcohol involvement determined by blood alcohol level);<br>Y91 (Evidence of alcohol involvement determined by level intoxication);<br>E244 (alcohol induced Pseudo-Cushing's syndrome);<br>E512 (Wernicke's Encephalopathy);<br>G312 (Degeneration of nervous system due to alcohol);<br>G621 (Alcoholic polyneuropathy);<br>G721 (Alcoholic myopathy);<br>I426 (Alcoholic cardiomyopathy);<br>K292 (Alcoholic gastritis);<br>K860 (Alcohol-induced chronic pancreatitis);<br>O354 (Maternal care for (suspected) damage to foetus from alcohol);<br>P043 (Foetus and newborn affected by maternal use of alcohol);<br>Q860 (Fetal alcohol syndrome (dysmorphic));<br>R780 (Finding of alcohol in blood);<br>T510 (Toxic effect of ethanol);<br>T511 (Toxic effect of methanol);<br>T519 (Toxic effect of alcohol; unspecified);<br>Y573 (Alcohol deterrents);<br>Z502 (Alcohol rehabilitation);<br>Z714 (Alcohol abuse counselling and surveillance);<br>Z721 (Alcohol Use).
**Alcohol-related deaths**	
Prior to 01/01/2000	
ICD-9 codes (deaths -primary cause)	4255 (alcoholic cardiomyopathy);<br>5710 (alcoholic fatty liver);<br>5711 (acute alcoholic hepatitis);<br>5712 (alcoholic cirrhosis of liver);<br>5713 (alcoholic liver damage; unspecified);<br>5714 (chronic hepatitis);<br>5715 (cirrhosis of liver without mention of alcohol);<br>5718 (other chronic nonalcoholic liver disease);<br>5719 (unspecified chronic liver disease without mention of alcohol);<br>E860 (accidental poisoning by alcohol; not elsewhere classified);<br>291 (alcoholic psychoses);<br>303 (alcohol dependence syndrome);<br>305 (non-dependent abuse of alcohol).
01/01/2000 onward	
ICD-10 codes (deaths-primary cause)	F10 (Mental & behavioural disorders due to use of alcohol); K70 (Alcoholic liver disease);<br>K73 (Chronic hepatitis; not elsewhere classified);<br>X45 (Accidental poisoning by and exposure to alcohol);<br>X65 (Intentional self-poisoning by and exposure to alcohol); Y15 (Poisoning by and exposure to alcohol; undetermined intent);<br>G312 (Degeneration of nervous system due to alcohol);<br>G621 (Alcoholic polyneuropathy); I426 (Alcoholic cardiomyopathy);

K292 (Alcoholic gastritis);
K740 (Hepatic fibrosis);
K741 (Hepatic sclerosis);
K742 (Hepatic fibrosis with hepatitic sclerosis); K746 (Other and unspecified cirrhosis of liver);
K860 (Alcohol induced chronic pancreatitis).

The ICD-codes here follow those used by Information Services Division NHS National Services Scotland and the National Records of Scotland at the time of data acquisition, for alcohol-related hospital episodes and alcohol-related deaths respectively.
Any pair of an *external alcohol code*[*] and *external code*[§] constitutes an alcohol-related episode.
[a] Combination of these codes in consecutive diagnoses listed constitutes an alcohol-related episode.
